# Evaluating Course Completion, Appropriateness, and Burden in the Understanding Multiple Sclerosis Massive Open Online Course: Cohort Study

**DOI:** 10.2196/21681

**Published:** 2021-12-07

**Authors:** Suzi B Claflin, Julie A Campbell, Kathleen Doherty, Maree Farrow, Barnabas Bessing, Bruce V Taylor

**Affiliations:** 1 Menzies Institute for Medical Research University of Tasmania Hobart Australia; 2 Wicking Dementia Research and Education Centre University of Tasmania Hobart Australia

**Keywords:** multiple sclerosis, massive open online course, health promotion, eHealth education, mobile phone

## Abstract

**Background:**

Massive open online course (MOOC) research is an emerging field; to date, most research in this area has focused on participant engagement.

**Objective:**

The aim of this study is to evaluate both participant engagement and measures of satisfaction, appropriateness, and burden for a MOOC entitled Understanding Multiple Sclerosis (MS) among a cohort of 3518 international course participants.

**Methods:**

We assessed the association of key outcomes with participant education level, MS status, caregiver status, sex, and age using summary statistics, and 2-tailed *t* tests, and chi-square tests.

**Results:**

Of the 3518 study participants, 928 (26.37%) were people living with MS. Among the 2590 participants not living with MS, 862 (33.28%) identified as formal or informal caregivers. Our key findings were as follows: the course completion rate among study participants was 67.17% (2363/3518); the course was well received, with 96.97% (1502/1549) of participants satisfied, with an appropriate pitch and low burden (a mean of 2.2 hours engagement per week); people living with MS were less likely than those not living with MS to complete the course; and people with a recent diagnosis of MS, caregivers, and participants without a university education were more likely to apply the material by course completion.

**Conclusions:**

The Understanding MS MOOC is fit for purpose; it presents information in a way that is readily understood by course participants and is applicable in their lives.

## Introduction

### Background

Massive open online course (MOOC) research is an emerging field [1,2]. The work done to date has focused on participant engagement, particularly course completion [3], which has presented a challenge for MOOCs because MOOCs have a mean 5% to 15% completion rate [4]. Few studies have evaluated course material appropriateness, participant satisfaction, and reasons for noncompletion. Here, we contribute to this ongoing conversation by evaluating the impact of education level, multiple sclerosis (MS) status, caregiver status, sex, and age on completion, satisfaction, perceived appropriateness, and burden of a MOOC on MS.

MOOCs emerged internationally into the knowledge economy in 2012, where they were heralded as a revolution that would democratize education by offering high-quality courses for free to anyone with access to an internet connection [5]. Since then, the number of MOOCs has rapidly increased; Class Central, the largest MOOC aggregator website, listed more than 13,000 MOOCs from more than 900 universities in 2019 [6]. However, despite the increased availability of MOOCS, these courses have struggled to reach and retain underserved students in the same numbers as their more privileged peers. As students from more affluent areas are more likely to participate in and complete MOOCs [7,8], MOOCs may exacerbate educational inequalities by offering additional resources to populations that also have access to a range of other educational opportunities.

Health and medicine MOOCs may encounter an additional challenge because they are often developed for use by people living with a health condition and their caregivers to address information asymmetry between the medical profession (as the suppliers) and people with health conditions and their caregivers (as consumers) [9-11]. However, because health status is related to socioeconomic status and education [12,13], people affected by a health condition may be less likely to enroll and complete a MOOC than those who are unaffected. Fortunately, previous studies suggest that these challenges can be addressed successfully. The Wicking Dementia Research and Education Centre (WDREC) has developed a MOOC on dementia that demonstrably improves knowledge of dementia in participants with a wide range of educational attainment [14,15], indicating that appropriately designed MOOCs can overcome some of these barriers.

Using the WDREC MOOCs as a successful model of knowledge dissemination, we have developed a free 6-week MOOC about MS [16] to increase awareness and understanding of MS in the MS community and interested laypeople. MS is a chronic autoimmune disorder where the immune system attacks and damages the central nervous system [17]. MS-related symptoms, such as mobility impairment and fatigue, may make it difficult for people living with MS to access traditional educational offerings [18]. After a year of development in collaboration with the MS community (eg, people with MS, carers, service providers, health care providers, and researchers), the Understanding MS MOOC was released in 2019 and had 2 open enrollments in that year. It was well received by participants, ranking first among the >2400 MOOCs released in 2019 based on participant reviews [6,19].

### Objective

In this study, we assessed the impact of the course on information asymmetry in the MS community. In health care (particularly in health services and health economics), information asymmetry (or asymmetry of information) relates to the difference in the information known by the consumer (eg, the patient or a member of the public) and that known by the producer or supplier, a health care professional [20]. In the information age, the gap creating information asymmetry could close if consumers can access appropriately pitched, validated, and targeted information sources [21]. Therefore, to assess the potential impact of the course on information asymmetry, we explored the overall course completion rate, participant satisfaction, perceived appropriateness and burden, and the association between these outcomes and demographic and health factors.

## Methods

### Overview

The data for this study were collected during the 2 enrollments of the Understanding MS MOOC administered in 2019. The course is free and available in English internationally on any internet-connected device (eg, computer or smartphone; [22]). Course content is presented in videos (transcripts are available for all videos), text, images, and animations. The content is presented in 6 modules over 6 weeks, and course participants can access the material for a total of 8 weeks. Each module contains at least 1 optional activity and discussion prompt. At the end of each module is a summary of the module content and a 10-question multiple-choice quiz. Participants can take the quiz as many times as they like but must achieve a score of 70% or higher to move on to the next module. The course covers topics ranging from the underlying pathology of MS to its impact on everyday life and includes both academic content and lived experience videos from a range of MS community members (for a more detailed description, refer to the study by Claflin et al [16]).

An optional feedback survey was accessible in the completion section during the 2- to 3-week period that the section was open before course closure. Therefore, the survey was only available to the participants who completed the course. We chose to place the feedback survey in the completion section to ensure that all survey respondents had completed the full intervention. An analysis of reasons for noncompletion is underway in a separate study. The feedback survey was adapted from a similar tool used to assess a WDREC MOOC about dementia [14] and queried participants’ overall satisfaction with the course and various aspects of the course. With a few exceptions, the questions in this survey were presented on a 5-point Likert scale, ranging from very dissatisfied to very satisfied, or strongly disagree to strongly agree and example survey questions are available in the study by Claflin et al [16].

Small changes to the web-based content were made between the 2 enrollments based on feedback from the first enrollment. We added 3 short videos (<3 minutes each): 1 on exercise physiology, 1 on physical therapy, and 1 on comorbidities. We added a couple of paragraphs of text about disease-modifying therapies and more clearly identified the activities in each module. We also added 2 small interactive features to help participants navigate through a series of short videos on symptoms and risk factors.

The course was advertised widely through social media, particularly through Facebook ads. Advertising targeted anglophone countries. Information about the course was also disseminated through the Menzies Institute network, as well as that of our project partners, Multiple Sclerosis Limited and WDREC, and other related organizations.

Participants in this study gave informed consent for their course-collected data, including their course feedback survey, to be used for research purposes in the introduction or orientation section of the course before they had access to any course content. This study was approved by the University of Tasmania Social Science Human Research Ethics Committee (H0017892).

### Demographic and Health Status Characteristics

This study evaluated 3 primary predictor variables for course completion and course satisfaction: MS status, caregiver status, and education level. These variables were of primary importance, as education has been shown to affect course completion in many MOOCs, and as a course intended for the MS community and interested laypeople, the course can only be considered fit for purpose if it is appropriate for people with MS and their caregivers.

Participants self-reported demographic and health status characteristics during course enrollment and in the feedback survey. This includes self-identification with various roles in the MS community. We categorized all participants into 2 MS status groups, as people with MS or those not living with MS, based on this information. We categorized people not living with MS into 2 caregiver status groups: not caregivers and caregivers, defined as anyone who identified as either a family or friend of a person with MS or a caregiver, thereby incorporating both formal and informal caregivers into a single group.

Similarly, participants self-reported their education level as grade 12 or below, occupational certificate or diploma, undergraduate degree, or postgraduate degree. We then categorized all participants into 2 education-level groups: no university education (grade 12 or below and occupational certificate or diploma) and university education (undergraduate or postgraduate degree), following the methodology of Goldberg et al [14].

Our secondary predictors were MS disease duration, sex, and age, which were self-reported during course enrollment. We calculated age from self-reported year of birth and calculated MS disease duration from self-reported year of diagnosis.

### Outcome Measures

#### Completion

We evaluated participant completion using course-collected data and compared the completion rate with the average for MOOCs, which is 5% to 15% [4]. We determined the course completion and final course module using quiz attempts. Any attempt to complete a quiz (whether or not the score was sufficient to move on to the next module) was considered an indication that the participant had completed the module. All module 6 (final module) quiz attempts were considered an indication of course completion. We assessed the association between completion rate and demographic and health status characteristics (MS status, caregiver status, education level, sex, age, and disease duration).

#### Satisfaction, Perceived Appropriateness, and Burden

We evaluated satisfaction, perceived appropriateness, and burden among course completers using data from the course feedback survey.

Satisfaction was determined using 2 questions. The first was a 5-point Likert scale that asked about overall satisfaction with the course. We categorized responses into 2 groups: satisfied (satisfied or very satisfied) or not satisfied (neutral, dissatisfied, or very dissatisfied). The second was a 6-point Likert scale that asked about the overall course quality. We categorized responses into 2 groups: average or below (average, poor, very poor), or above average (good, very good, and excellent).

We evaluated the appropriateness and burden of the course with questions querying (1) self-reported agreement that the participant could understand the content, (2) that the language was too technical, (3) that there was too much or too little material, (4) that the course improved their understanding, (5) that the material could improve care or quality of life for people with MS, (6) that they would recommend the course, and (7) that they had already applied course material in their lives. Responses were categorized into 2 groups: agree (agree or strongly agree) and disagree (neutral, disagree, or strongly disagree). We also assessed the burden by comparing the self-reported average time spent on a single course module between groups.

### Analysis

We cleaned the data set by removing any staff accounts and removing the second attempts of any participant who took part in both enrollments. During data cleaning, we designated ages (based on self-reported year of birth) of <10 years or >95 years as no data, as these values were deemed implausible. Similarly, we excluded impossible or uninterpretable years of diagnosis (eg, 1 or 1900).

As this data set was overpowered, there were many statistically significant differences that were not of interest because they were not reflective of materially significant differences between groups. To account for this, we set a threshold of material significance for comparisons between categorical variables, which required a 5% difference between groups. We report the results of these materially significant differences (all of which are statistically significant). As age and disease duration were continuous variables, we evaluated their effects on all outcomes of interest. To determine whether the enrollments could be evaluated together, we compared the outcomes of interest to assess if there were any materially significant differences (>5%).

We assessed the demographics of study participants using the sample size and percentage of the cohort for categorical variables and mean and SD for continuous variables. We assessed the association between the predictor variables on the responses of interest and the relationships between the predictor variables using 2-tailed chi-square and *t* tests. As disease duration was not normally distributed, we evaluated its association with the outcomes of interest using the Mann-Whitney rank-sum tests of equal medians. We used Pearson correlation to evaluate the association between the average time taken to complete a module and participant age and disease duration. In all analyses, statistical significance was set at *P*<.05. All analyses were conducted using STATA (version 16.0, StataCorp).

## Results

### Participant Characteristics

**Figure 1:**
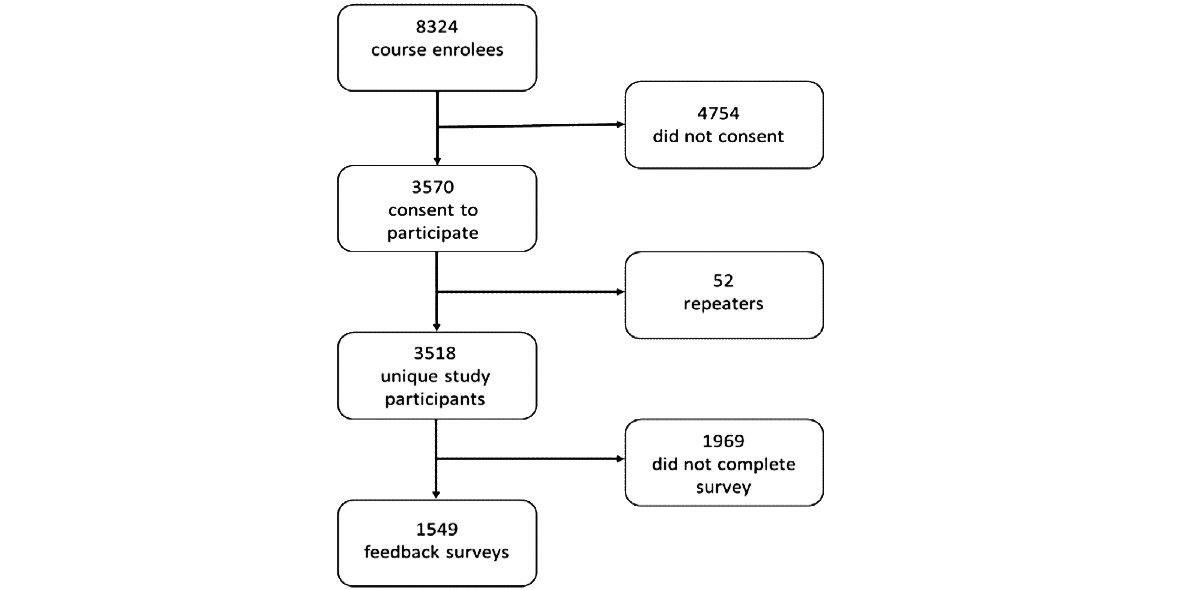
Inclusion flowchart.

In total, 8324 people enrolled in the first 2 enrollments of the Understanding MS MOOC, 3912 (46.99%) of whom completed the course. After removing 52 second attempts, 3518 unique participants across the 2 course enrollments gave permission for their data to be used in research; 1549 consenting course completers also completed a feedback survey (Figure 1).

Participant characteristics are presented in Table 1. Differences between enrollments were <5% for the outcomes of interest. Therefore, data from both enrollments were assessed together.

The majority of the participants were women and had an undergraduate degree or higher education level (course data in Table 1). Nearly two-thirds of the participants resided in Australia, with other large anglophone countries with high MS prevalence comprising the other most well-represented nations in the sample (eg, Canada and New Zealand). Nearly a third of the participants were people with MS. Of the 2096 course participants not living with MS, 862 (41.12%) identified as formal or informal (family or friends of people with MS) caregivers.

**Table 1:** Characteristics of study participants who provided course-collected data and course completers who supplied feedback surveys. Please note that participants could select multiple MS^a^ community roles^b^.

Characteristic			Course-collected data		Feedback survey
**Gender, n (%)**			3492 (100)		1412 (100)
	Male	552 (15.81)		227 (16.08)	
	Female	2940 (84.19)		1185 (83.92)	
**Education level, n (%)**			2876 (100)		1549 (100)
	No university education	1169 (40.65)		595 (38.41)	
	University education	1707 (59.35)		954 (61.59)	
**MS community roles^c^, n (%)**			3024 (100)		1549 (100)
	Person with MS	928 (30.69)		437 (28.21)	
	Family member or friend	664 (21.96)		382 (24.66)	
	Carer	352 (11.64)		139 (8.97)	
	Service provider	360 (11.9)		92 (5.94)	
	Allied health	815 (26.95)		365 (23.56)	
	General practitioner	67 (2.22)		41 (2.65)	
	Neurologist	62 (2.05)		18 (1.16)	
	Advocate	67 (2.22)		44 (2.84)	
	Researcher	123 (4.07)		44 (2.84)	
	Other or no MS community role	374 (12.37)		321 (20.72)	
**Country of residence, n (%)**			3509 (100)		1417 (100)
	Australia	2180 (62.13)		907 (64.01)	
	Canada	100 (2.85)		38 (2.68)	
	United Kingdom	255 (7.27)		101 (7.13)	
	Ireland	133 (3.79)		44 (3.11)	
	New Zealand	277 (7.89)		127 (8.72)	
	United States	106 (3.02)		35 (2.47)	
	South Africa	51 (1.45)		27 (1.91)	
	Other	407 (11.6)		138 (9.74)	
**Final section completed, n (%)**			3518 (100)		—^d^
	<Module 1	620 (17.62)		—	
	Module 1	251 (7.13)		—	
	Module 2	90 (2.59)		—	
	Module 3	74 (2.1)		—	
	Module 4	72 (2.05)		—	
	Module 5	48 (1.36)		—	
	Module 6	2363 (67.17)		—	
Age (years), mean (SD)			44.38 (13.34)^e^		46.78 (13.10)^f^
Disease duration (years), median (SD)			4 (10)		5 (10)^g^

^a^MS: multiple sclerosis.

^b^Among people with multiple sclerosis, approximately half of the participants who provided course-collected data and those who provided feedback data had a disease duration of 4 years or less. Consequently, the distribution was highly skewed toward 0 years (diagnosis in 2019; Multimedia Appendix 1).

^c^Multiple selections possible.

^d^Not available.

^e^N=3292.

^f^N=1330.

^g^N=401.

### Completion

Of the 3518 course participants who gave permission for their course-collected data to be used in research, 2363 (67.17%) completed the course. There were significant differences in the completion rate between MS status groups (*χ^2^_1_*=36.8; *P*<.001; Table 2). People with MS were less likely to complete the course than those not living with MS (539/928, 58.08% compared with 1455/2096, 69.42%). People with MS completed an average of 3.9 modules while those not living with MS completed an average of 4.5. This association was consistent across all course modules, with people with MS less likely to complete module 1 (*χ^2^_1_*=26.7; *P*<.001), module 2 (*χ^2^_1_*=22.2; *P*<.001), module 3 (*χ^2^_1_*=26.03; *P*<.001), module 4 (*χ^2^_1_*=28.8; *P*<.001), and module 5 (*χ^2^_1_*=27.9; *P*<.001).

**Table 2:** The percentage of participants completing the course, satisfied with the course, or agreeing with various statements about the course in the course feedback survey in different participant groups, and the absolute difference between groups. Italicized values indicate materially significant (>5%) differences between groups.

Participant groups			Course completion (%)		Course feedback survey data (%)																				
					Satisfied		Above average quality		Improved understanding		Could understand		Language too technical		Too much material		Not enough material		Can improve care		Can improve quality of life		Would recommend		Already applied
**University education, n (%)**																									
	No	744 (63.64)		582 (97.82)		583 (98.31)		562 (96.1)		572 (97.28)		47 (8.06)		28 (4.79)		69 (11.82)		551 (94.03)		528 (90.26)		572 (98.28)		391 (66.95)	
	Yes	1162 (68.07)		920 (96.44)		936 (98.42)		874 (93.6)		919 (98.08)		65 (6.96)		48 (5.17)		152 (16.34)		856 (92.04)		829 (89.72)		899 (96.98)		554 (61.15)	
	|x-y|^a^(%)	4.43		1.38		0.11		2.49		0.80		1.10		0.37		4.53		1.98		0.54		1.30		*5.80^b^*	
**MS^c^ status, n (%)**																									
	People with MS	539 (58.08)		420 (96.11)		421 (97)		387 (89.79)		418 (96.76)		32 (7.44)		22 (5.14)		67 (15.55)		383 (89.07)		369 (87.03)		413 (96.72)		281 (66.59)	
	People not living with MS	1455 (69.42)		1082 (97.3)		1098 (98.92)		1049 (96.42)		1073 (98.17)		80 (7.36)		54 (4.98)		154 (14.22)		1024 (94.29)		988 (91.06)		1038 (97.78)		664 (62.17)	
	|x-y| (%)	*11.34^b^*		1.19		1.91		*6.62^b^*		1.41		0.08		0.16		1.33		*5.22^b^*		4.03		1.06		4.42	
**Caregiver status, n (%)**																									
	No	877 (71.07)		672 (97.39)		678 (98.55)		646 (95.99)		666 (98.09)		49 (7.25)		33 (4.9)		101 (15.05)		630 (93.75)		610 (90.77)		654 (97.32)		388 (58.7)	
	Yes	578 (67.05)		410 (97.16)		420 (99.53)		403 (97.11)		407 (98.31)		31 (7.54)		21 (5.12)		53 (12.86)		394 (95.17)		378 (91.53)		404 (98.54)		276 (67.81)	
	|x-y| (%)	4.02		0.23		0.98		1.12		0.22		0.29		0.21		2.19		1.42		0.75		1.22		*9.11^b^*	
**Sex, n (%)**																									
	Female	1985 (67.52)		1149 (96.96)		1166 (98.56)		1098 (94.33)		1150 (98.29)		73 (6.28)		48 (4.13)		166 (14.27)		1086 (93.38)		1043 (89.99)		1127 (97.41)		728 (63.69)	
	Male	366 (66.30)		221 (97.36)		221 (98.22)		210 (95.45)		210 (95.02)		26 (11.76)		21 (9.63)		39 (17.73)		200 (91.74)		196 (90.74)		214 (97.72)		139 (64.65)	
	|x-y| (%)	1.21		0.39		0.34		1.12		3.27		*5.48^b^*		*5.51^b^*		3.45		1.64		0.75		0.36		0.96	

^a^|x-y|: absolute difference between groups.

^b^Materially significant difference level was set at >5%.

^c^MS: multiple sclerosis.

To further explore this, we evaluated the completion rate among participants who completed module 1 and found that the difference between those living with MS and those not living with MS shrank to about 6% (539/707, 76.24% compared with 1455/1762, 82.58%). This difference was maintained among those who completed module 2. Among module 3 completers, the difference between people with MS and those not living with MS dropped below the threshold for material significance and continued to decline in the remaining module completion groups. There were no materially significant differences in completion based on caregiver status, sex, or education level, but age was significantly associated with completion. Course completers were more likely to be older than noncompleters (Table 3). However, the effect size was not large; the mean age of completers was 45 years compared with 42 years for noncompleters. Similarly, among people with MS, participants with more recent diagnoses were less likely to complete the course than those who had been living with MS for longer periods (Table 3). However, the effect size was small (median disease duration of 1 year compared with 2 years).

**Table 3:** Results of *t* tests, Mann-Whitney rank-sum tests of equal medians, and Pearson correlations evaluating the association between age and disease duration, and all outcome variables.

Participant groups		Course completion	Course feedback survey data												Average hours to complete 1 module^a^		
			Satisfied	Above average quality	Improved understanding	Could understand	Language too technical	Too much material	Not enough material	Can improve care	Can improve quality of life	Would recommend	Already applied	Coefficient		*P* value	
**Age**														0.06		*.03^b^*	
	*t* test	−6.26 (3290)	−0.052 (1328)	−0.32 (1325)	−1.55 (1304)	−0.52 (1308)	4.55 (1301)	4.17 (1299)	2.38 (1301)	0.16 (1298)	0.42 (1292)	−1.41 (1293)	1.27 (1277)				
	*P* value	*<.001^b^*	.60	.75	.12	.60	*<.001^b^*	*<.001^b^*	*.02^b^*	.87	.67	.16	—^c^				
**Multiple sclerosis disease duration**														−0.023		.65	
	z	−2.154	−0.304	0.551	2.349	0.231	−0.083	0.694	2.391	2.027	0.865	0.158	3.078				
	*P* value	*.03^b^*	.77	.59	*.02^b^*	.82	.93	.49	*.02^b^*	*.04^b^*	.39	.88	*.002^b^*				

^a^Estimates from Pearson correlation.

^b^Indicate *P* values <.05.

^c^Not available.

### Satisfaction, Perceived Appropriateness, and Burden

Overall, course completers were satisfied with the course, with 96.97% (1502/1549) of those completing the feedback survey reporting that they were satisfied or very satisfied (Multimedia Appendix 2). They also rated the quality of the course highly, with 98.38% (1519/1544) rating it above average (good, very good, or excellent). The pitch of the course appears appropriate, with nearly all participants agreeing that they could understand the information, that the course improved their understanding, and that they would recommend the course.

Participants also found the material helpful, with 63.42% (945/1490) reporting that they had applied information from the course at course completion, and nearly all) agreed that the information could improve care (1407/1516, 92.81%) or quality of life (1357/1509, 89.93%) for people with MS. In addition, the burden was low (average of 2.2 hours to complete a module). Only 5.02% (76/1513) agreed that there was too much material, whereas 14.6% (221/1514) agreed that there was too little material.

There were few materially significant differences in the responses of the demographic and health status groups (Table 2). People with MS were less likely to report improved understanding because of the course material (*χ^2^_1_*=26.2; *P*<.001) and were less likely to agree that the course material could improve care (*χ^2^_1_*=12.6; *P*<.001). Among people not living with MS, caregivers were more likely to report applying the course material by course completion than noncaregivers (*χ^2^_1_*=60.0; *P*<.001).

University education was also associated with applying the course material; participants with a university education were less likely to report applying the course material at course completion than those without one (*χ^2^_1_*=5.2; *P*=.02). Sex was significantly associated with agreement that the language in the course was too technical (*χ^2^_1_*=8.4; *P*=.004) and that there was too much material (*χ^2^_1_*=11.7; *P*<.001). Male participants were more likely to agree with these statements than female participants. However, there was no difference in the average time spent per module between males and females.

Age was associated with several outcomes of interest (Table 3). Participants who agreed that there was too much material in the course were more likely to be older (mean age of 47 years compared with 40 years). Correspondingly, those who agreed that there was not enough material were more likely to be younger. Participants who agreed that the language was too technical were also more likely to be older. However, the effect sizes for the latter 2 associations were small, with differences in mean age between 2.5 and 3.5 in the 2 groups. Similarly, increasing age was associated with a greater average number of hours taken to complete a module, but the effect size was small (coefficient=0.02; Table 3).

Among people with MS, disease duration was also associated with several outcomes of interest (Table 3). People with MS who agreed that the course had improved their understanding were more likely to have a shorter disease duration than those who did not (median disease duration of 1 year compared with 4 years). A total of 93.5% (172/184) of participants with disease durations of ≤4 years reported improved understanding, compared with 86.2% (181/210) of those with disease durations of >4 years. Similarly, participants who reported that they had applied information from the course by course completion were more likely to be recently diagnosed (median of 1 year compared with 3 years). A total of 73.9% (136/184) participants with a disease duration of ≤4 years reported applying the course material, compared with 58.9% (46/78) of the participants with a disease duration >4 years.

Participants with more recent diagnoses were also more likely to report that there was not enough material in the course than those with older diagnoses (median disease duration of 1 year compared with 2 years) and that the content of the course could improve care for people with MS (median of 1 year compared with 2 years), although the effect sizes of these comparisons were small (1 year).

### Associations Between Demographic and Health Status Characteristics

Among course completers, education level was associated with MS status, caregiver status, and sex (Table 4). People with MS were less likely than participants not living with MS (230/437, 52.63% compared with 724/1112, 65.11%) to have a university education. Among those not living with MS, caregivers were less likely than noncaregivers (215/422, 50.95% compared with 509/690, 73.77%) to have a university education. Males were more likely than females to have university education (158/227, 69.6%) compared with 61.01% (723/1185).

Age was significantly associated with caregiver status and education level (Table 4). Participants who were caregivers were more likely to be older than noncaregivers (mean age 50 years compared with 44 years). Similarly, participants without a university education were more likely to be older than those with a university education (mean of 50 years compared with 45 years). Among people with MS, MS disease duration was not associated with sex or education level but was strongly associated with age (Table 4).

**Table 4:** Results of chi-square and *t* tests evaluating the associations between demographic and health status groups.

Participant groups		University education			MS^a^ status					Caregiver status						Sex			Age, coefficient (*P* value)^b^
		No	Yes	People with MS		People not living with MS		No			Yes								
**MS status^c^, n (%)**																			
	People with MS	207 (47.37)	230 (52.63)	—^d^		—		—				—		—			—		
	People not living with MS	388 (34.89)	724 (65.11)	—		—		—				—		—			—		
**Caregiver status^e^, n (%)**																			
	No	181 (26.23)	509 (73.77)	—		—		—				—		—			—		
	Yes	207 (49.05)	215 (50.95)	—		—		—				—		—			—		
**Sex^f^, n (%)**																			
	Female	462 (38.99)	723 (61.01)	350 (29.54)		835 (70.46)		513 (61.44)			322 (38.56)			—			—		
	Male	69 (30.40)	158 (69.60)	69 (30.40)		158 (69.60)		107 (67.72)			51 (32.28)			—			—		
**Age**																			
	*t* test	6.05 (1328)			−1.80 (1328)		—		−7.15 (934)				—		0.93 (1323)			—	
	*P* value	*<.001^g^*			.07		—		*<.001^g^*				—		.35			—	
**Disease duration^h^**																			
	z	0.533			—		—		—				—		−0.556			0.461	
	*P* value	.59			—		—		—				—		.58			*<.001^g^*	

^a^MS: multiple sclerosis.

^b^Estimates from Pearson correlation.

^c^*χ^2^_1_*=20.6; *P*<.001

^d^Not available.

^e^*χ^2^_1_*=60.03; *P*<.001

^f^University education: *χ^2^_1_*=5.99; *P*=.01; multiple sclerosis status: *χ^2^_1_*=0.07; *P*=.80; caregiver status: *χ^2^_1_*=2.2; *P*=.14

^g^Indicate *P* values <.05.

^h^Among people with multiple sclerosis.

## Discussion

### Principal Findings

To our knowledge, the Understanding MS web-based course is the largest MS-related web-based course in the world. To date, more than 13,000 people from 128 countries have enrolled in the course, and it was ranked first among the >2400 courses released in 2019 based on participant reviews. Correspondingly, we found that overall, the Understanding MS MOOC had a completion rate that was more than 3 times higher than the average for MOOCs and very high participant satisfaction. However, there were materially significant differences in participant experience among the different participant groups. People with MS were less likely than those not living with MS to complete the course. Although 63.42% (945/1490) of all course completers reported applying the course material, caregivers and those without a university education were more likely to apply it. Overall, the Understanding MS MOOC is fit for purpose, with an appropriate pitch and burden level, and presents information that is relevant to participants’ lives. By disseminating relevant content directly to information consumers (people with MS, caregivers, and those without a university education), the course addresses information asymmetry in the MS community.

### Completion

The first 2 open enrollments of the Understanding MS MOOC had an average completion rate of 47% (data not presented here). However, among study participants (the subset of all course participants who consented to take part in this research), there was a 67.17% (2363/3518) completion rate. This is 3-9 times higher than the average for all MOOCs, which fluctuates between 5% and 15% [4]. Course completion was about 11% higher among those not living with MS (539/928, 58.08%) than among those with MS (1455/2096, 69.42%), driven by noncompletion early in the course, particularly in module 1. This may be because of the additional challenges faced by people with MS that may interfere with their ability to complete the course, including complications arising from MS-related symptoms such as fatigue and cognitive impairment.

In addition, course completion was about 4% higher among participants with a university education (1162/1707, 68.07%) than among those without (744/1169, 63.64%). Although this difference is not materially significant, it is larger than other similar courses, such as the Understanding Dementia MOOC developed by WDREC [4], who observed a difference of 0.44% between groups. This discrepancy may be because of the underlying differences in the course participants. The Understanding Dementia MOOC is intended primarily for dementia carers rather than those living with dementia, whereas the Understanding MS MOOC is aimed at a broad audience, including people with the condition; 30.69% (928/3024) of this sample comprised people with MS. People with MS were both less likely to have completed university and less likely to complete the Understanding MS MOOC. The difference in completion between education levels may reflect the difference in completion rate associated with MS status. The data support the possibility that education level and health status interactively affect completion. Among the study participants, people with MS without a university education had the lowest completion rate (250/450, 55.56%) of any MS status or education level group. Conversely, people not living with MS who had a university education had the highest completion rate (904/1278, 70.74%).

### Satisfaction, Perceived Appropriateness, and Burden

Among course completers, satisfaction and perceived appropriateness were high in all demographic and health status groups, with >98% satisfied and ≥95% agreeing that they could understand the course material. This agrees with previous work on health and medicine MOOCs, which found >80% participant satisfaction among allied health professionals (Harvey et al 2014 [13]) and members of the community (Tieman et al 2018 [23]). The course also presents a low burden for participants, with participants reporting that the material took an average of 2.2 hours per week to complete. This is far lower than the average 4.2 hours per week required by health and medicine MOOCs [24].

Almost two-thirds of the course completers reported applying course material by completion. However, there were significant differences in the application of course materials between the participant groups. People with MS who were newly diagnosed, caregivers, and those without a university education were more likely to report that they had applied the course material. Newly diagnosed people with MS, who were also more likely to report that the course improved their understanding, were well positioned to apply the course material immediately. Among people not living with MS, caregivers may be better positioned to apply the material immediately. Again, the association between caregiver status and education level, with caregivers less likely to have a university education than noncaregivers, may in part drive the observed association between education level and application of course material. The data support this; caregivers without a university education were the most likely to report applying information by completion (140/201, 69.65%) of any caregiver or education level group. Noncaregivers with university education were least likely to report applying it (279/483, 57.76%). However, this result also agrees with the large body of work demonstrating that higher education levels are associated with higher health literacy and better health outcomes [13,25]. Participants with lower education levels may have lower baseline health literacy and MS-related knowledge, and therefore, learn more from the course.

### Knowledge Dissemination to Address Information Asymmetry

Health information is a valuable commodity. High-value health care relies on effective information exchange [11], and better information dissemination is needed to close the gap between health information providers and information consumers [10]. This study demonstrates that participation in the Understanding MS MOOC helps to address information asymmetry among course completers. By course completion, participants successfully translated information by applying it to their lives. This is particularly clear among newly diagnosed people with MS (disease duration of 0-4 years) and caregivers, who are the most likely to apply the course material by completion (136/182, 74.73% and 276/407, 67.81%) reported applying course material by completion, respectively). Recent research suggests that services intended for caregivers need to be sensitive to the fluctuating demands placed upon caregivers and be flexible in their support [23]. This study shows that the Understanding MS MOOC accommodates the needs of health information consumers, such as newly diagnosed and caregivers, and can help to address information asymmetry in the MS community.

### Strengths, Limitations, and Future Directions

The main strength of this study is the large and diverse course evaluation cohort. The study participants comprised an international cohort of MS community members and interested laypeople with a range of educational attainment. This study had 2 main limitations. First, the analysis grouped formal and informal carers. These groups may have different needs and characteristics that we were unable to parse in this study. Second, the course evaluation survey was only presented to course completers, making the group vulnerable to selection bias. Although we cannot control for this bias, we have presented our results accordingly. Future research should explore the impact of MS status on the reasons for noncompletion.

### Conclusions

The Understanding MS MOOC is an accessible health education intervention with a pitch and burden that is appropriate for course participants. It presents information relevant to the lives of the participants and can be immediately applied. Because a large proportion of course participants identify with MS community roles that are traditional consumers of information, the results of this study suggest that the Understanding MS courses can help to address information asymmetry.

## Appendix

Multimedia Appendix 1 Disease duration (years) among people with multiple sclerosis who participated in this study (n=825).

Multimedia Appendix 2 Percent agreement among course feedback survey participants in response to various statements about the course (exact percentage provided in data labels).

## Abbreviations

MOOC: massive open online course
MS: multiple sclerosis
WDREC: Wicking Dementia Research and Education Centre
