# Supplementary figures and images for: Evaluating Course Completion, Appropriateness, and Burden in the Understanding Multiple Sclerosis Massive Open Online Course: Cohort Study
Source: J Med Internet Res. 2021 Dec 7;23(12):e21681. doi: 10.2196/21681 (PMC8693196; doi:10.2196/21681)

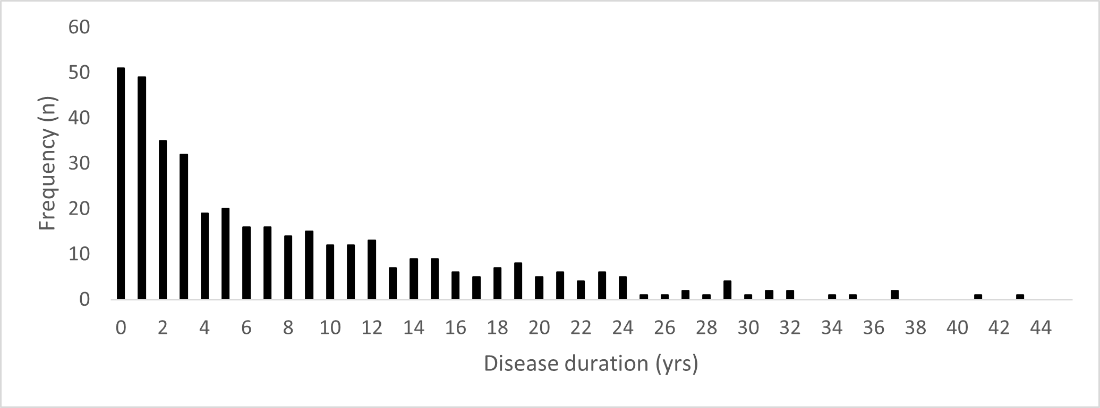

Supplement: Multimedia Appendix 1 [file jmir_v23i12e21681_app1.png]

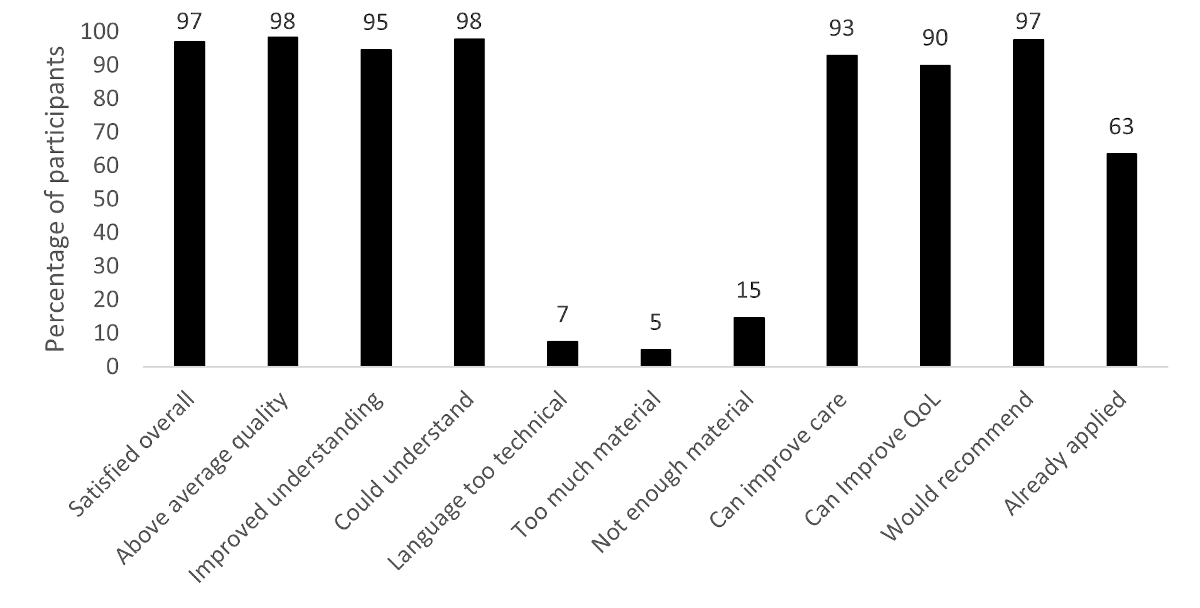

Supplement: Multimedia Appendix 2 [file jmir_v23i12e21681_app2.png]
